# Bioinformatic Analysis of Contrasting Expression Patterns and Molecular Interactions of TIMPs in Breast Cancer: Implications for Tumor Progression and Survival

**DOI:** 10.3390/pathophysiology33010013

**Published:** 2026-02-02

**Authors:** Lorena Cayetano-Salazar, Jhactcidi Jackeline García-López, Dania A. Nava-Tapia, Eymard Hernández-López, Caroline Weinstein-Oppenheimer, Julio Ortiz-Ortiz, Marco Antonio Leyva-Vázquez, Miguel Ángel Mendoza-Catalán, Adán Arizmendi-Izazaga, Napoleón Navarro-Tito

**Affiliations:** 1 Laboratorio de Biología Celular del Cáncer, Facultad de Ciencias Químico Biológicas, Universidad Autónoma de Guerrero, Av. Lázaro Cárdenas S/N, Chilpancingo 39090, Guerrero, Mexico; lorenacayetano@uagro.mx (L.C.-S.); jhactcidijackeline.gl@gmail.com (J.J.G.-L.); danianavatapia@uagro.mx (D.A.N.-T.); eymardh7@gmail.com (E.H.-L.);; 2 Postgraduate and Research, TecNM-TESOEM, Paraje de San Isidro S/N, La Paz 56400, State of Mexico, Mexico; 3 Escuela de Química y Farmacia, Facultad de Farmacia, Universidad de Valparaíso, Av. Gran Bretaña 1093, Playa Ancha, Valparaiso CP 2360102, Chile; caroline.weinstein@uv.cl; 4 Laboratorio de Investigación en Metabolismo y Cáncer, Facultad de Ciencias Químico Biológicas, Universidad Autónoma de Guerrero, Av. Lázaro Cárdenas S/N, Ciudad Universitaria, Colonia La Haciendita, Chilpancingo 39090, Guerrero, Mexico; julioortiz@uagro.mx; 5 Laboratorio de Biomedicina Molecular, Facultad de Ciencias Químico Biológicas, Universidad Autónoma de Guerrero, Av. Lázaro Cárdenas S/N, Ciudad Universitaria, Colonia La Haciendita, Chilpancingo 39090, Guerrero, Mexico; leyvamarco13@gmail.com (M.A.L.-V.); mamendoza@uagro.mx (M.Á.M.-C.)

**Keywords:** breast cancer, TIMPs, tumor progression, biomarkers, bioinformatic analysis

## Abstract

**Background/Objectives:** Although tissue inhibitors of metalloproteinases (TIMPs) are key regulators in breast cancer, their differential expression, clinical relevance, and molecular roles remain unclear. This study aimed to compare the expression patterns of the four TIMPs in breast cancer and evaluate their molecular interactions and associated pathways through an integrated bioinformatic analysis. **Methods:** The expression of TIMPs and their correlations with MMPs were analyzed using the TCGA PanCancer, cBioPortal, and GEO datasets. Associations between TIMP expression and overall survival were assessed in the TCGA Breast Invasive Carcinoma PanCancer cohort. Pathway enrichment analysis was performed using GO, KEGG, and DAVID. The relationships between immune cell infiltration, stromal cells, and TIMP expression were assessed using the EPIC algorithm. Statistical analyses were performed using R. **Results:** *TIMP1* was the only inhibitor overexpressed in breast tumors and showed significant associations with the Luminal B, HER2, TNBC, and normal-like subtypes, along with a modest increase across stages. *TIMP2*, *TIMP3*, and *TIMP4* were downregulated in tumors. High expression of *TIMP1* and *TIMP4* correlated with better overall survival. *TIMP1*-associated genes were enriched in NF-kappa and PI3K–Akt signaling and actin cytoskeleton components. *TIMP2* was linked to Hedgehog and MAPK pathways and actin-related elements. *TIMP3* correlated with Hedgehog and PI3K–Akt signaling, DNA damage response, and membrane components. TIMP4 was associated with VEGF, MAPK, PI3K–Akt, DNA damage pathways, and actin organization. *TIMP2* showed strong positive correlations with *MMP2* and *MMP14*, while *TIMP4* showed negative correlations with *MMP1* and *MMP9*. Interestingly, we found a strong positive correlation between *TIMP2* and *TIMP3* with *ADAM12*, as well as between *TIMP2* and *TIMP3* with *ADAM10*, and negative correlations with *ADAM15*. The differential expression of TIMPs favors greater infiltration of immune cells related to tumor progression and poor prognosis in breast cancer patients. **Conclusions:** TIMPs display contrasting expression profiles and distinct pathway associations in breast cancer. *TIMP1* emerges as the only consistently overexpressed inhibitor, while *TIMP4* appears as a promising prognostic marker with unique MMP correlations that may influence tumor behaviors.

## 1. Introduction

Worldwide, breast cancer remains the most common malignancy and the second leading cause of cancer-related deaths in women [1]. Breast cancer is a heterogeneous disease with different histopathological and biological features [2]. According to immunohistochemical markers, including the presence or absence of estrogen receptors (ERs), progesterone receptors (PRs), and human epidermal growth factor receptor 2 (HER2), breast cancer can be classified into molecular subtypes. These subtypes are Luminal A (ER- and PR-positive, HER2-negative, low Ki67), Luminal B (ER- and/or PR-positive, HER2-positive or high Ki67), HER2-enriched (ER- and PR-negative, HER2-positive), and triple-negative (TNBC) (ER-, PR-, and HER2-negative) [3]. Each molecular subtype shows different responses to clinical therapy and requires different treatment strategies [4]. In addition to molecular and histological subtypes, breast cancer is evaluated according to tumor grade and size, lymph node status, and metastasis [5]. The TNM staging system (tumor/node/metastasis) for breast cancer is a critical tool for assessing tumor size and the extent of spread. It provides information on the involvement of regional lymph nodes and indicates whether the cancer has metastasized to distant sites in the body [6]. Therefore, the TNM classification system represents a fundamental tool in oncology, guiding clinical decision-making and the selection of optimal therapeutic strategies for breast cancer patients [7].

Novel potential biomarkers related to breast cancer development and progression have recently been proposed, including tissue inhibitors of metalloproteinases (TIMPs) have been proposed [8]. TIMPs regulate tissue extracellular matrix turnover, inhibit matrix metalloproteinases (MMPs), induce apoptosis, inhibit tumor cell migration, invasion, and angiogenesis, and mainly inhibit MMPs [9]. TIMPs are 21 to 28 kDa proteins that bind to MMPs and reversibly block their activity. Four TIMPs (TIMP-1, TIMP-2, TIMP-3, TIMP-4) have been described; all are present in the extracellular matrix (ECM) in soluble form, except TIMP-3, which is bound to the ECM [10,11]. The inhibitory function of TIMPs is related to the presence of a C-terminal and an N-terminal domain, each with three conserved disulfide bonds, where the N-terminal domain folds into itself and binds to the active site of MMPs to inhibit their activity [11,12,13].

Matrix metalloproteinases (MMPs), which are produced by non-malignant stromal cells, as well as by tumor cells, are widely expressed in breast cancer across multiple cell types that constitute the tumor microenvironment. These enzymes contribute to tumor progression by regulating cell growth, invasion, angiogenesis, and metastasis [14]. The MMPs involved in promoting tumor progression include the collagenases MMP1 and MMP13, the gelatinases MMP2 and MMP9, the stromelysins MMP3 and MMP11, the matrilysin MMP7, the metalloelastase MMP12, and the membrane-type MMP MMP14 [15].

In addition to inhibiting MMPs, tissue inhibitors of metalloproteinases (TIMPs) can also suppress the activity of members of the A Disintegrin and Metalloproteinase (ADAM) family [8]. ADAMs can activate membrane-bound growth factors, cytokines, receptors, and adhesion molecules, which in turn can trigger signaling pathways such as EGFR/HER2, TNF-α, and Notch, thereby promoting proliferation, invasion, angiogenesis, epithelial–mesenchymal transition (EMT), immune evasion, and therapeutic resistance. ADAM-8, ADAM-9, ADAM-10, ADAM-12, ADAM-15, and ADAM-17 have been associated with the initiation and progression of breast cancer [16].

Structurally, TIMPs are similar and can selectively inhibit different MMPs; however, they do not do so with the same efficacy [11]. In particular, TIMP-1 preferentially inhibits MMP-7, MMP-9, MMP-1, and MMP-3, whereas TIMP-2 more effectively inhibits MMP-2. TIMP-3 can inhibit MMP-2 and MMP-9, whereas TIMP-4 inhibits the catalytic activity of MMP-14 and MMP-2 [17,18,19]. Therefore, an imbalance between TIMPs and MMPs is an important factor for cancer development, as it affects the integrity of the extracellular matrix [11].

Bioinformatic analyses have indicated TIMP-2 as a potential prognostic factor in several cancer types, as it participates in pathways associated with ECM regulators, ECM degradation, and ECM disassembly [20]. Likewise, in another bioinformatics analysis, it was shown that TIMP-2 downregulates several breast cancer subtypes; moreover, TIMP-2 expression was associated with overall survival with different clinical features. Thus, TIMP-2 could serve as a potential target and prognostic biomarker in breast cancer [21]. However, there is little information on the biological roles of the TIMPs in breast cancer. Therefore, in this study we aim to elucidate the relationships of TIMPs with breast cancer progression stages, probability of disease-free survival, interaction with target proteins, correlation of TIMPs with MMPs, correlation of immune cell infiltrates with TIMPs, and epigenetic factors regulating TIMP expression.

We found that *TIMP1* is overexpressed in breast cancer patients, with increased expression observed in the more aggressive molecular subtypes and in advanced disease stages. In contrast, *TIMP2*, *TIMP3*, and *TIMP4* were underexpressed in tumor tissue. Our results demonstrate that high expression of *TIMP1* and TIMP4 is associated with improved overall survival in breast cancer patients. KEGG and GO analyses revealed that genes positively correlated with *TIMP1* were enriched in the NF-kappa and PI3K-Akt signaling pathways and in cytoskeletal components. *TIMP2* showed positive correlations and enrichment in Hedgehog and MAPK signaling pathways and in actin cytoskeleton components. *TIMP3* was positively correlated and enriched in Hedgehog and PI3K-Akt signaling pathways, DNA damage response genes, and cellular membrane components. *TIMP4* exhibited positive correlations and enrichment in VEGF, MAPK, and PI3K-Akt signaling pathways, as well as in DNA damage response genes and actin cytoskeleton components. We also observed that *TIMP1*, *TIMP2*, and *TIMP3* were positively correlated with MMPs. Notably, among all TIMPs, *TIMP4* was the only one negatively correlated with *MMP1*, *MMP9*, and *MMP13*. Interestingly, *TIMP2* and *TIMP3* showed strong positive correlations with *ADAM12* and *ADAM10*.

Furthermore, it is well established that tumor-infiltrating immune cells, classified as lymphoid or myeloid, can either promote or suppress tumor growth by modulating immune responses through B cells, CD8+ cytotoxic T cells, CD4+ helper T cells, and regulatory T cells, while natural killer (NK) cells exert cytotoxic activity to target cancer cells [22]. Myeloid cells are composed of macrophages with M1 (antitumor) and M2 (protumor) phenotypes, antigen-presenting dendritic cells (DCs), and myeloid-derived suppressor cells (MDSCs), which function to suppress immunity. The balance between protumor and antitumor immune states determines the degree of cancer progression and therapeutic response in patients diagnosed with breast cancer [23]. Recent studies have focused on identifying novel pathways and biomarkers that enhance the immunogenic activity of dendritic cells (DCs) within the tumor microenvironment. In this context, TIMP-1 has recently emerged as a potential functional immune biomarker [24], in addition to its canonical role as a metalloproteinase inhibitor. Similarly, recent findings highlight that TIMPs increase the infiltration and survival of CD8+ T cells and are positively correlated with CD4+ T cells, CD8+ T cells, macrophages, neutrophils, and dendritic cells [25,26,27]. This study revealed that the expression of *TIMP3*, *TIMP2*, and *TIMP1* is positively correlated with the infiltration of cancer-associated fibroblasts (CAFs), endothelial cells, macrophages, and NK cells. Conversely, the expression of *TIMP4*, *TIMP3*, *TIMP2*, and *TIMP1* is negatively correlated with the infiltration of B cells, CD4^+^ T cells, and CD8^+^ T cells. Interestingly, it was observed that both high and low TIMP expression favors the differential infiltration of immune cells, such as B cells, CD4^+^ T cells, CD8^+^ T cells, macrophages, NK cells, CAFs, endothelial cells, and other cellular infiltrates. Our results demonstrate that TIMPs can have antitumor or protumor effects, depending on the breast cancer context, molecular subtype, stage, and the involvement of the tumor microenvironment, such as immune system infiltrates.

## 2. Materials and Methods

### 2.1. TIMP Expression Levels in Breast Cancer Patients and Cell Lines

For the bioinformatic analyses, gene expression data were obtained from the Breast Invasive Carcinoma TCGA PanCancer dataset [28] accessed via the cBioPortal for Cancer Genomics (http://cbioportal.org) on 10 November 2025 [29]. Expression data for *TIMP1*, *TIMP2*, *TIMP3*, and *TIMP4* were downloaded as mRNA expression Z-scores relative to normal tissue samples. For the analysis of TIMP expression in molecular subtypes of breast cancer, the dataset includes 1084 samples in total, of which 499 samples correspond to Luminal A samples, 197 to Luminal B samples, 78 to HER2+ samples, 171 to basal triple-negative samples, and 36 to normal-like samples. Molecular subtype classification was performed according to the PAM50 gene expression signature assay, and only samples from female patients with valid PAM50 classification were included. For the analysis of gene expression according to tumor stage, only samples from patients with complete clinical data on tumor stage classification, based on the American Joint Committee on Cancer (AJCC) Tumor Stage Code, were considered. The dataset comprised 277 stage I, 628 stage II, 137 stage III, and 39 stage IV samples. For both molecular subtype and tumor stage analyses, Z-scores > 2 and Z-scores < −2 were defined as high and low expression, respectively.

TIMP expression validation was performed in patient samples and breast cancer cell lines. Expression analysis in patients and breast cancer cell lines was performed using the GSE45827 and GSE48213 datasets, respectively, which were obtained from the Gene Expression Omnibus GEO public repository (https://www.ncbi.nlm.nih.gov/geo/) on 4 January 2026. Gene expression data for patient samples and cell lines were obtained from the Affymetrix GeneChip Human Genome U133A and Affymetrix GeneChip Human Exon 1.0 ST arrays. The classification of molecular subtypes in breast cancer patients and cell lines was performed and managed as described in the datasets. A total of 41 breast cancer samples were included: basal TN, 30 HER2+, 29 Luminal A, and 30 Luminal B, as well as 11 normal tissue samples. In addition, 14 cell lines were classified as Luminal A, 6 as Luminal B, 7 as HER2+, 10 as basal-like/TNBC, and 5 as normal-like. The levels of TIMP1, TIMP2, TIMP3, and TIMP4 proteins in breast cancer patient samples compared to normal or non-tumor samples were analyzed in patient biopsies obtained from The Human Protein Atlas (https://www.proteinatlas.org/) on 6 January 2026. The difference in TIMP levels was categorized based on staining intensity: negative, low, medium, and high. Immunohistochemistry was performed using the HPA053417 antibody for TIMP1 and the CAB010203 antibody for TIMP2. Eleven breast tumor tissue samples were used for TIMP1, ten for TIMP2, and three normal samples. It is important to note that protein expression data for TIMP3 and TIMP4 are still unavailable.

### 2.2. Overall Survival Analysis of TIMP Expression in Breast Cancer Patients

For survival analysis in breast cancer patients, clinical and survival phenotypic data were collected from the Breast Invasive Carcinoma TCGA PanCancer dataset [28] (accessed on 11 November 2025), which includes 1084 breast cancer patient samples. Kaplan–Meier (K–M) survival analyses were subsequently performed, followed by log-rank tests, using the “survival” package in the R software (version 4.3.2). The primary objective was to assess differences in overall survival (OS) between groups characterized by low and high expression levels of *TIMP1*, *TIMP2*, *TIMP3*, and *TIMP4*. Univariate Cox regression models were applied to estimate hazard ratios (HRs) and 95% confidence intervals (CIs). To evaluate the prognostic value of TIMPs, Cox proportional hazards regression analyses were performed. Univariate Cox regression models were applied to estimate hazard ratios (HRs) and 95% confidence intervals (CIs). Statistical analyses were performed using the survival package in the R software (version 4.3.2). Survival curves were generated using the survminer and ggplot2 packages.

### 2.3. Functional Enrichment Analysis of TIMPs

Genes showing positive and negative correlations with each TIMPs were subjected to GO and KEGG enrichment analyses using the functional annotation tools available on the DAVID website (https://bio.tools/david_bioinformatics_resources) [30], accessed on 13 January 2025. The resulting data were organized and visualized using various bubble plots. Datasets were filtered using a significance threshold of *p* < 0.05. Functional categorization was performed into Biological Processes (BPs), Cellular Components (CCs), Molecular Function (MF), and Kyoto Encyclopedia of Genes and Genomes (KEGG). DAVID enrichment analysis characterizes the functional relevance of a gene set and evaluates its involvement in specific pathways and biological processes through the exploration of BP, MF, CC, and KEGG annotations. Additionally, the analysis provided information on gene counts and their relevance within pathways, along with their functional classifications. This approach enabled a comprehensive understanding of the functions and implications of the selected genes, particularly in the context of cellular components and processes related to invasion and metastasis in cancer, thereby enhancing overall data interpretation and highlighting their biological significance.

### 2.4. Correlation Matrix: TIMPs vs. MMPs and ADAMs

A Spearman correlation matrix was constructed to assess the associations between the gene expression levels of each TIMP and those of matrix metalloproteinases (*MMP1*, *MMP2*, *MMP3*, *MMP8*, *MMP9*, *MMP13*, *MMP14*, and *MMP16*), as well as with the expression ADAMs (*ADAM8*, *ADAM9*, *ADAM10*, *ADAM12*, *ADAM15*, and *ADAM17*). Gene expression data were obtained from the Breast Invasive Carcinoma TCGA PanCancer dataset [28], accessed on 18 December 2025, and downloaded as mRNA expression Z-scores relative to normal tissue samples. Analyses were conducted using the R software (version 4.3.2), employing the “Hmisc” and “ggplot2” packages to generate the correlation matrix.

### 2.5. Immune Infiltration Analysis and Stratified Comparison According to TIMP Expression

Immune cell infiltration in breast cancer samples was inferred using the EPIC deconvolution algorithm. Normalized RNA-seq expression data (TPM values) from the Breast Invasive Carcinoma TCGA PanCancer dataset (*n* = 1084) were used as input to estimate the relative proportions of major immune and stromal cell populations, including macrophages, CD4^+^ T cells, CD8^+^ T cells, B cells, NK cells, cancer-associated fibroblasts (CAFs), and endothelial cells. To assess the global associations between *TIMP1*, *TIMP2*, *TIMP3*, and *TIMP4* expression levels and immune infiltration, Spearman correlation analyses were performed between TIMP gene expression and the estimated cell-type fractions. Correlation coefficients and corresponding *p*-values were calculated, and results were visualized using a correlation heatmap. In addition, samples were stratified into high- and low-expression groups for each TIMP based on the median expression value. Differences in immune and stromal cell infiltration between high and low TIMP expression groups were evaluated for each cell type. Statistical significance was assessed using the Wilcoxon rank-sum test, followed by Benjamini–Hochberg false discovery rate (FDR) correction to account for multiple testing. These results were visualized using boxplots to illustrate differences in immune cell infiltration according to TIMP expression status. All analyses were conducted using the R software (version 4.4.1), and data processing and visualization were performed using the “EPIC”, “Hmisc”, “statix”, “ggplot2”, “ggpubr”, and “pheatmap” packages.

### 2.6. Statistical Analysis

For analysis of the expression levels of *TIMP1*, *TIMP2*, *TIMP3*, and *TIMP4* in the molecular subtypes, tumor stages of breast cancer and cell lines, one-way ANOVA was performed, followed by Bonferroni and Dunnet post hoc tests, with statistical significance set at *p* < 0.05. For overall survival analysis, Kaplan–Meier (K–M) plots were generated, and log-rank tests were applied. Cox proportional hazards models (HRs) were also used, with *p*-values < 0.05 considered statistically significant. For GO and KEGG enrichment analyses, a global Spearman correlation analysis was performed to identify genes positively and negatively correlated with each TIMP. Similarly, Spearman correlations were used to construct the correlation matrix between each TIMP, MMP and ADAM. All statistical analyses were conducted using the R software (version 4.3.2).

## 3. Results

### 3.1. TIMP Expression Levels and Correlation with Patient Progression Stages and Breast Cancer Cell Lines

**Figure 1:**
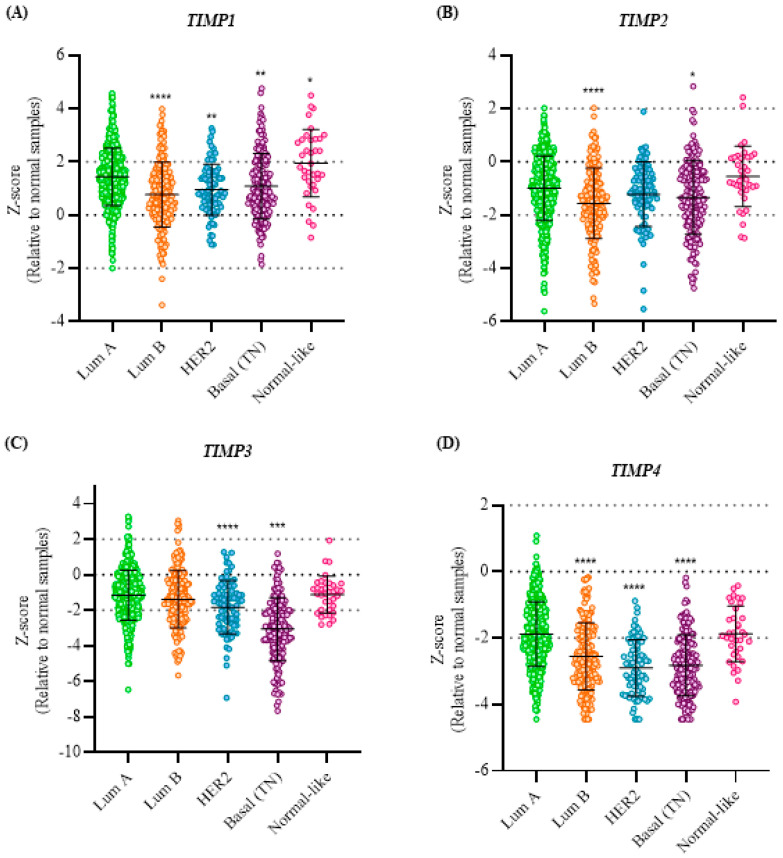
Expression levels of TIMPs across breast cancer molecular subtypes. Violin plots showing the relative expression (Z-score) of TIMP genes across breast cancer molecular subtypes: Luminal A (*n* = 499), Luminal B (*n* = 197), HER2+ (*n* = 78), basal (TN) (*n* = 171), and normal-like (*n* = 36). (**A**) *TIMP1* gene expression. (**B**) *TIMP2* gene expression. (**C**) *TIMP3* gene expression. (**D**) *TIMP4* gene expression. Data were obtained from the TCGA PanCancer Breast Invasive Carcinoma dataset via cBioPortal for Cancer Genomics. Gene expression values are represented as Z-scores relative to normal tissue samples. Statistical analyses were performed using one-way ANOVA, followed by Bonferroni post hoc tests to compare gene expression levels across molecular subtypes relative to the Luminal A subtype. Statistical significance relative to Luminal A samples is indicated as *p* < 0.05 (*), *p* < 0.01 (**), *p* < 0.001 (***), and *p* < 0.0001 (****).

TIMPs play roles in several biological functions, such as regulating MMP activation, angiogenesis, cell growth, apoptosis, and metastasis [9,10,11,12]. For this reason, it is important to determine TIMP expression levels in breast cancer molecular subtypes, considering that each molecular subtype is distinct and is associated with the treatment response and prognosis. Expression levels of *TIMP1*, *TIMP2*, *TIMP3*, and *TIMP4* were analyzed in 981 breast cancer samples from the TCGA PanCancer Breast Invasive Carcinoma dataset, obtained via cBioPortal. The dataset used for analysis of TIMP expression across molecular subtypes included 499 Luminal A, 197 Luminal B, 78 HER2+, 171 basal triple-negative, and 36 normal-like samples. Our results show that *TIMP1* was overexpressed across the different molecular subtypes of breast cancer patients. However, higher expression was observed only in the Luminal A and normal-like subtypes compared with Luminal B, HER2+, and basal (TN) subtypes. The normal-like subtype showed a significant increase in expression compared with the Luminal A subtype (*p* < 0.05) (Figure 1A). In the Luminal B, HER2+, and basal (TN) subtypes, *TIMP1* showed a significant decrease in expression compared with the Luminal A subtype (*p* < 0.01 and *p* < 0.0001) (Figure 1A).

Our analysis revealed that *TIMP2*, *TIMP3*, and *TIMP4* are underexpressed across the different molecular subtypes of breast cancer. Specifically, *TIMP2* showed a significant decrease in expression in the Luminal B and basal (TN) subtypes compared with Luminal A (*p* < 0.0001 and *p* < 0.05) (Figure 1B). *TIMP3* expression was significantly decreased in the HER2+ and basal (TN) subtypes relative to Luminal A (*p* < 0.001 and *p* < 0.0001) (Figure 1C). *TIMP4* showed a significant decrease in the Luminal B, HER2+, and basal (TN) subtypes (*p* < 0.0001) (Figure 1D). Our analysis suggests that TIMPs expression varies depending on the breast cancer subtype, which could have implications for tumor progression and potential therapeutic strategies depending on the molecular subtype of breast cancer. Breast cancer can progress rapidly from early to advanced stages. The earliest stage is stage 0 (carcinoma in situ), followed by stages I to IV; at the latter stage, the breast cancer has metastasized to other organs [31]. The rapid progression of breast cancer makes early diagnosis and monitoring of cancer progression difficult [32]. We determined the gene expression levels of *TIMP1*, *TIMP2*, *TIMP3*, and *TIMP4* according to tumor stage in breast cancer patients. The analysis included 277 samples from patients with stage I, 628 samples with stage II, 137 samples with stage III, and 39 samples with stage IV tumors. Gene expression is presented as Z-scores relative to normal tissue samples.

**Figure 2:**
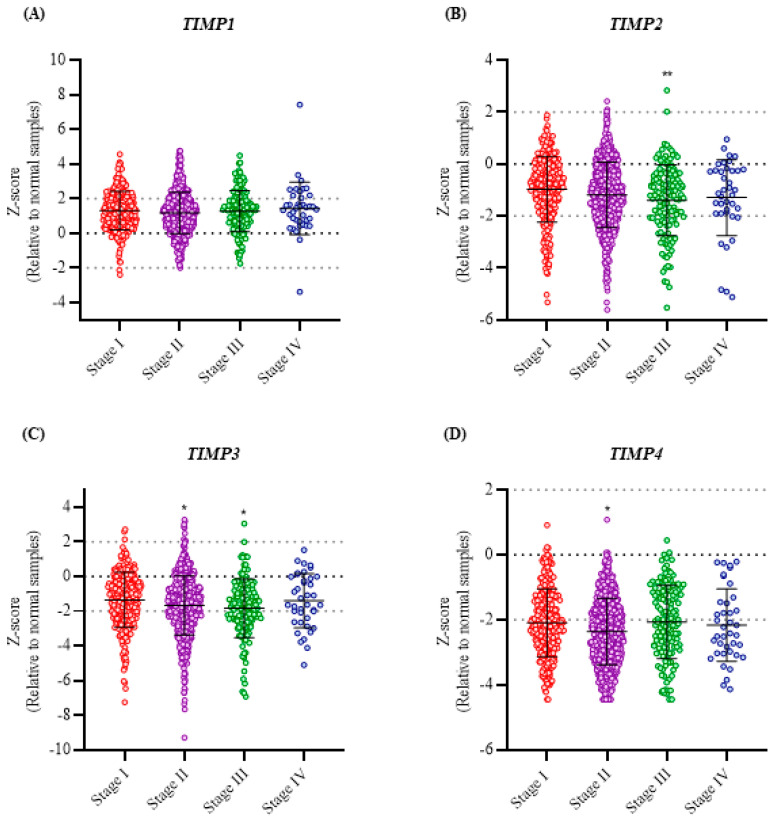
Expression levels of TIMPs across breast cancer tumor stages. Analysis of *TIMP1* (**A**), *TIMP2* (**B**), *TIMP3* (**C**), and *TIMP4* (**D**) gene expression in breast tumor samples classified by tumor stage from I to IV. Values are expressed as Z-scores relative to normal tissue samples. Violin plots show the distribution and density of the data within each group. Statistical analyses were performed using one-way ANOVA followed by Bonferroni post hoc tests to compare expression levels across tumor stages relative to stage I. Statistical significance relative to stage I is indicated as *p* < 0.05 (*) and *p* < 0.01 (**).

*TIMP1* was the only TIMP found to be overexpressed in breast cancer patients. However, *TIMP1* expression did not show significant changes across the different tumor stages (Figure 2A). Analysis of *TIMP2* revealed a significant decrease in expression at stage III compared with stage I (*p* < 0.01) (Figure 2B). In contrast, *TIMP3* showed a trend toward decreased expression in advanced stages, with significant reductions at stages II and III compared with stage I (*p* < 0.01) (Figure 2C). Finally, *TIMP4* expression was significantly reduced at stage II compared with stage I (*p* < 0.01) (Figure 2D).

**Figure 3:**
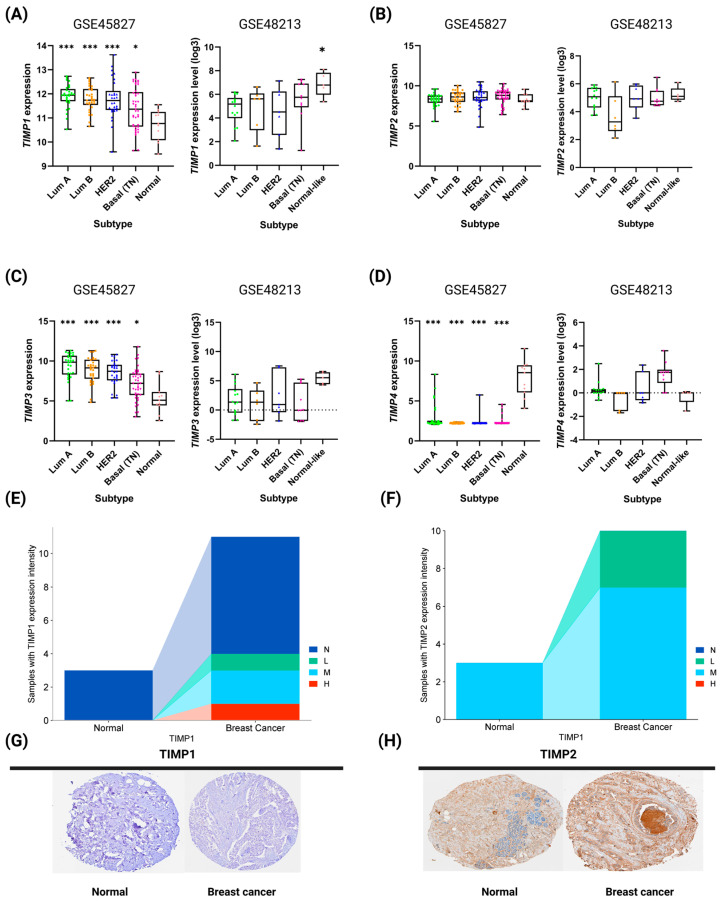
TIMP expression levels in different molecular subtypes of breast cancer patient samples and cell lines. Gene expression analysis of *TIMP1* (**A**), *TIMP2* (**B**), *TIMP3* (**C**), and *TIMP4* (**D**). Dataset GSE45827 shows the corresponding TIMP expression in different molecular subtypes of breast tumor samples. Dataset GSE4813 shows TIMP expression in cell lines classified into different molecular subtypes of breast cancer. Statistical analyses were performed using one-way ANOVA followed by Dunnett’s tests to compare expression levels across tumor stages. Statistical significance is indicated as *p* < 0.05 (*), *p* < 0.001 (***). The frequency of breast cancer and normal samples showing TIMP1 and TIMP2 protein expression, negative (N), low (L), medium (M), and high (H), are represented in sections (**E**,**F**). Sections (**G**,**H**) show representative images of TIMP1 and TIMP2 in breast cancer and normal samples, respectively.

TIMP expression was validated in patient samples classified according to the different molecular subtypes of breast cancer, as well as in cell lines representative of these subtypes. It is important to note that, unlike the previous analysis—which compared the normalized gene expression of TIMPs exclusively between tumor and normal samples—this analysis included normal samples and did not use them to normalize the expression of the subtypes. *TIMP1* and *TIMP3* expression was observed to decrease significantly and progressively as breast cancer progressed to more aggressive molecular subtypes (Figure 3A,C from dataset GSE45827). In contrast, *TIMP4* expression was significantly lower in all molecular subtypes compared to normal samples (Figure 3D from dataset GSE45827). *TIMP2*, in contrast, showed no significant differences in expression among the different molecular subtypes analyzed (Figure 3B from dataset GSE45827).

Additionally, analysis of TIMP expression in cell lines corresponding to different breast cancer subtypes (Figure 3) revealed an expression pattern consistent with that observed in the TCGA PanCancer Breast Invasive Carcinoma dataset (Figure 1). In particular, *TIMP1* expression was significantly higher in cell lines classified as normal-like compared to the Luminal A, Luminal B, HER2+, and basal (triple-negative) subtypes (Figure 3A–D from dataset GSE48213). However, at the protein level, no significant differences in *TIMP1* and *TIMP2* expression were detected between breast cancer samples and normal tissue (Figure 3E,F). It is important to note that, to date, the Human Protein Atlas includes no data reporting the protein expression of TIMP3 and TIMP4. While these results provide interesting exploratory findings, they should be interpreted with caution until experimental evidence from a larger number of samples is available.

### 3.2. Survival Analysis of TIMP Expression in Breast Cancer Patients

**Figure 4:**
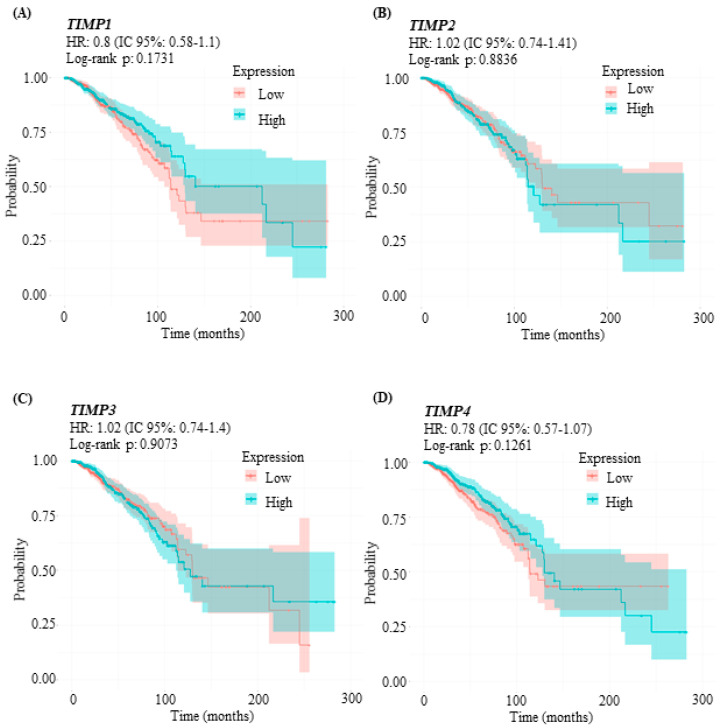
Expression levels of TIMPs and their correlation with overall survival (OS) in breast cancer patients. Red lines represent low TIMP expression, and blue lines represent high TIMP expression. The Y-axis represents the probability of survival, and the X-axis represents patient follow-up time. A representative *p*-value is shown in each graph. Univariate Cox regression models were applied to estimate hazard ratios (HRs) and 95% confidence intervals (CIs). (**A**) Correlation between *TIMP1* expression and OS; (**B**) correlation between *TIMP2* expression and OS; (**C**) correlation between *TIMP3* expression and OS; and (**D**) correlation between *TIMP4* expression and OS.

The role of TIMPs in breast cancer is very controversial; there are reports describing that TIMPs may have both a protumor and antitumor effect. It has been suggested that elevated levels of TIMPs are associated with early relapse in breast cancer patients [33]. Considering that *TIMP1* expression is increased in breast cancer—unlike *TIMP2*, *TIMP3*, and *TIMP4*, whose expression is decreased—we performed a Kaplan–Meier analysis of overall survival using data collected from TCGA (Figure 4). Comparisons between breast cancer patient groups with high and low TIMPs expression were conducted using Kaplan–Meier survival curves, log-rank tests, and Cox regression models. Although the results were not statistically significant, our analysis showed that high *TIMP1* expression tended to be associated with improved overall survival, with a hazard ratio (HR) of 0.8 (95% CI: 0.58–1.1). Analysis of *TIMP2* expression revealed no significant association with overall survival, HR = 1.02 (95% CI: 0.74–1.41, *p* = 0.8836). Similarly, *TIMP3* expression was not significantly associated with overall survival; HR = 1.02 (95% CI: 0.74–1.4, *p* = 0.9073). In contrast, high *TIMP4* expression showed a trend toward improved overall survival in breast cancer patients, with HR = 0.78 (95% CI: 0.57–1.07, *p* = 0.1261).

### 3.3. Correlation of TIMPs with KEGG and GO Signaling Pathways Related to Biological Processes, Cellular Components, and Molecular Functions in Breast Cancer

**Figure 5:**
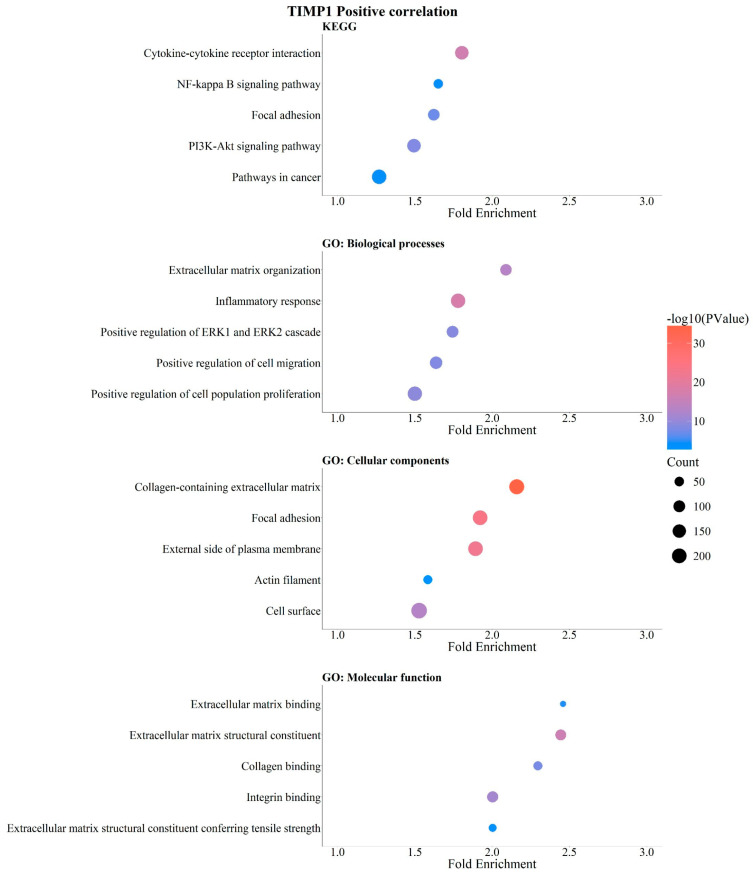
Positive correlation of *TIMP1* with breast cancer-related genes. GO and KEGG enrichment analysis (https://bio.tools/david_bioinformatics_resources, accessed on 11 November 2025). Categorization into Biological Processes (BPs), Cellular Components (CCs), Molecular Functions (MFs), and Kyoto Encyclopedia of Genes and Genomes (KEGG). Data were organized and are presented as bubble plots. Only data with statistical significance (*p* < 0.05) were included.

Breast cancer is associated with molecular alterations in genes that regulate mechanisms related to migration and invasion in breast cancer [34,35]. Using the DAVID web tool, we performed an analysis of TIMP gene expression levels that showed a positive correlation with genes enriched in KEGG signaling pathways, Biological Processes (BPs), Cellular Components (CCs), and Molecular Functions (MFs). Values of *p* < 0.050 were considered statistically significant. *TIMP1* expression was positively correlated and enriched in signaling pathways related to cytokine–receptor interaction, NF-kappa B signaling, focal adhesion, and PI3K-Akt signaling, as well as cancer-related pathways, all of which are associated with breast cancer progression (Figure 5). In addition, analysis of genes involved in biological processes revealed significant enrichment in extracellular matrix organization, inflammatory response, positive regulation of cell migration and proliferation, and the ERK1/ERK2 pathway (Figure 5). Furthermore, *TIMP1* showed positive correlation with cellular components of the collagen-containing extracellular matrix, focal adhesion-related components, plasma membrane components, actin cytoskeleton, and cell surface structures (Figure 5). *TIMP1* expression levels were also positively correlated with molecular functions associated with extracellular matrix constituents, collagen binding, integrin binding, and structural functions of the extracellular matrix (Figure 5).

**Figure 6:**
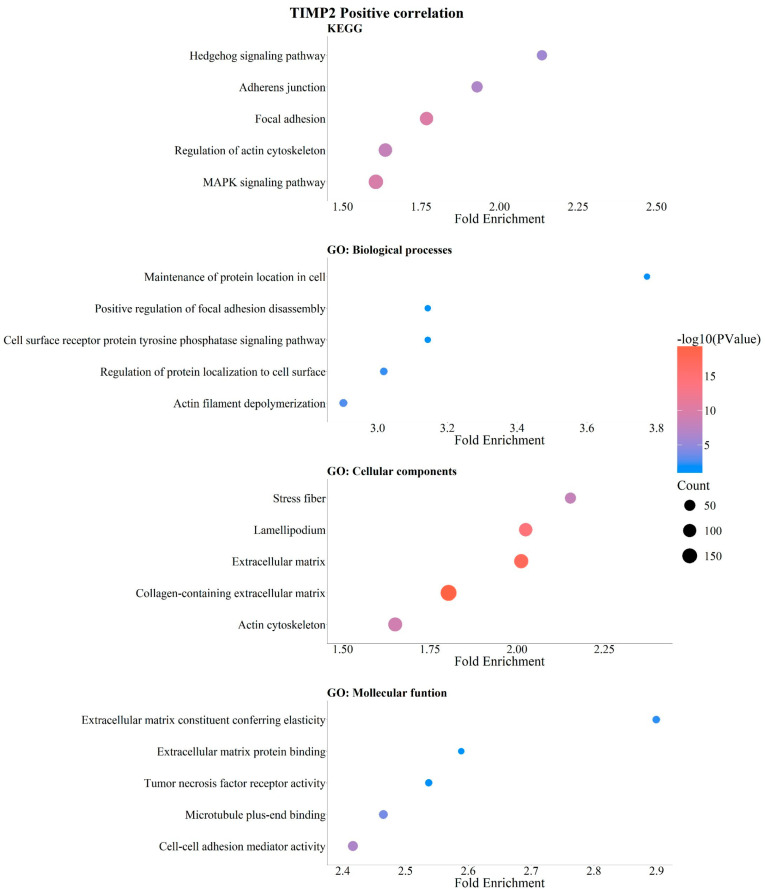
Positive correlation of *TIMP2* with KEGG signaling pathways and GO enrichment in breast cancer. KEGG and GO analysis (https://bio.tools/david_bioinformatics_resources, accessed on 11 November 2025). Categorization into Biological Processes (BPs), Cellular Components (CCs), Molecular Functions (MFs), and Kyoto Encyclopedia of Genes and Genomes (KEGG). Data were organized and are presented as bubble plots. Only data with statistical significance (*p* < 0.05) were included.

Analysis of *TIMP2* expression levels revealed positive correlation and enrichment in Hedgehog signaling, adherens junctions, focal adhesion, actin cytoskeleton regulation, and MAPK signaling pathways (Figure 6). Analysis of biological processes enriched by *TIMP2* showed positive regulation of genes involved in focal adhesion disassembly, receptor-type protein tyrosine phosphatase signaling, regulation of protein localization at the cell surface, and actin filament depolarization (Figure 6). GO analysis of cellular component genes positively correlated with *TIMP2* identified stress fiber components, lamellipodia, extracellular matrix components, collagen-containing matrix components, and actin cytoskeleton elements (Figure 6). Analysis of molecular functions revealed that genes positively associated with *TIMP2* expression are involved in enhancing cellular elasticity, extracellular matrix binding, microtubule binding, and mediating cell adhesion activity (Figure 6). Overall, our analysis suggests that *TIMP2* expression levels are positively correlated with genes involved in cellular signaling, migration, and invasion processes in breast tumor cells.

**Figure 7:**
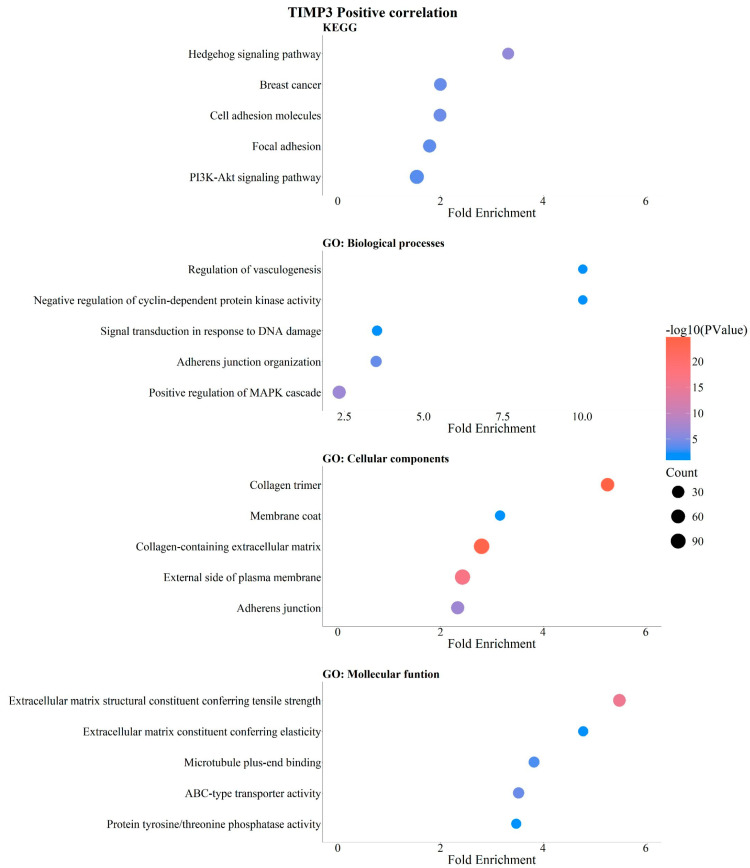
Positive correlation of *TIMP3* with KEGG signaling pathways and GO enrichment in breast cancer. KEGG and GO analysis (https://bio.tools/david_bioinformatics_resources, accessed on 11 November 2025). Categorization into Biological Processes (BPs), Cellular Components (CCs), Molecular Functions (MFs), and Kyoto Encyclopedia of Genes and Genomes (KEGG). Data were organized and are presented as bubble plots. Only data with statistical significance (*p* < 0.05) were included.

The analysis revealed a positive correlation between *TIMP3* expression levels and enrichment in Hedgehog signaling, breast cancer-related pathways, cell adhesion molecules, focal adhesion, and PI3K-Akt signaling (Figure 7). Analysis of biological processes showed that *TIMP3* expression positively regulates genes involved in vasculogenesis, negative regulation of cyclin-dependent protein kinases, adherens junction organization, and positive regulation of MAPK signaling. Interestingly, *TIMP3* also showed positive correlation with genes involved in DNA damage response signaling (Figure 7). GO analysis of cellular components positively correlated with *TIMP3* identified extracellular matrix components, including collagen trimers, membrane coatings, and adherens junctions (Figure 7). Analysis of molecular functions revealed genes positively associated with *TIMP3* that contribute to extracellular matrix elasticity, microtubule binding, ABC-type transport activity, and protein phosphatase activity (Figure 7).

**Figure 8:**
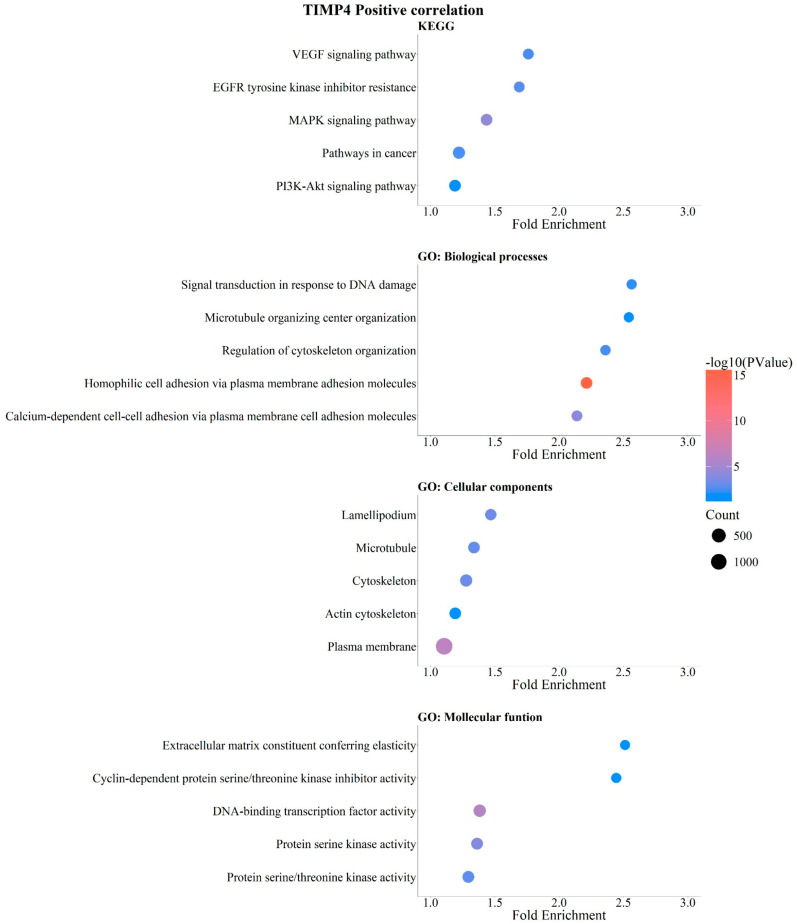
Positive correlation of *TIMP4* with KEGG signaling pathways and GO enrichment in breast cancer. KEGG and GO analysis (https://bio.tools/david_bioinformatics_resources, accessed on 11 November 2025). Categorization into Biological Processes (BPs), Cellular Components (CCs), Molecular Functions (MFs), and Kyoto Encyclopedia of Genes and Genomes (KEGG). Data were organized and are presented as bubble plots. Only data with statistical significance (*p* < 0.05) were included.

The analysis revealed a positive correlation between *TIMP4* expression levels and enrichment in VEGF signaling, resistance to EGFR tyrosine kinase inhibitors, breast cancer-related pathways, MAPK signaling, and PI3K-Akt signaling (Figure 8). Biological processes enriched in association with *TIMP4* expression included genes involved in DNA damage response signaling, microtubule organization, cytoskeleton organization regulation, and cell membrane adhesion molecules (Figure 8).

GO analysis of cellular components positively correlated with *TIMP4* identified lamellipodia, microtubules, actin cytoskeleton, and plasma membrane components (Figure 8). In terms of molecular functions, genes positively associated with *TIMP4* included extracellular matrix constituents contributing to elasticity, kinase activity, and DNA-binding transcription factor activity (Figure 8). Our analysis suggests that *TIMP4* is positively correlated with genes regulating signaling pathways that promote a more aggressive phenotype in breast cancer, as well as genes associated with the actin cytoskeleton, cell adhesion, and the formation of membrane structures related to increased cellular migration and invasion capacity (Figure 8).

### 3.4. Correlation Analysis of TIMPs with MMPs

**Figure 9:**
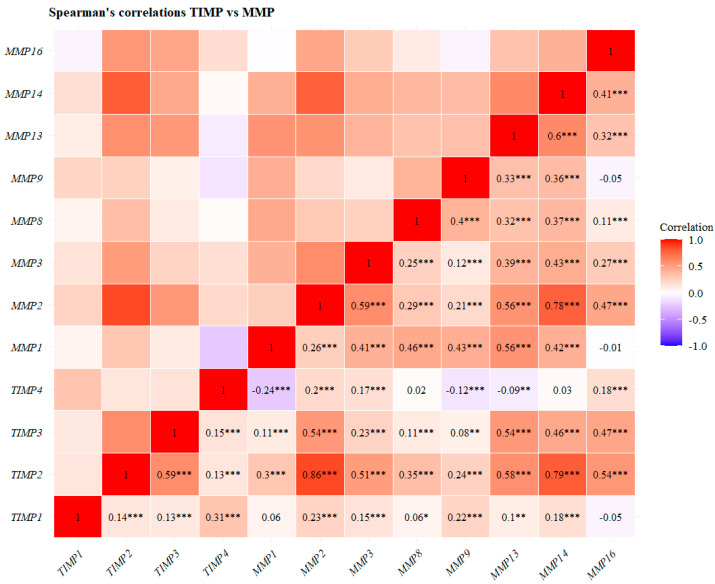
Spearman correlation matrix between TIMPs and matrix metalloproteinase (MMP) gene expression levels in invasive breast cancer. Results are presented as mRNA expression Z-scores relative to normal samples. Correlations were calculated using the Spearman coefficient with the “Hmisc” package in R (version 4.3.2). Statistical significance levels are reported as *p* < 0.05 (*), *p* < 0.01 (**), and *p* < 0.001 (***). Colors range from red (positive correlation) to blue (negative correlation).

TIMPs have been associated with breast cancer invasion and metastasis due to their ability to bind to MMPs and prevent their activation process [11]. We performed a Spearman correlation matrix between TIMP and MMP gene expression levels. Our analysis revealed statistically significant correlations between TIMPs and MMPs. For *TIMP1*, we observed positive correlations with *MMP2*, *MMP3*, *MMP9*, and *MMP14* (*p* < 0.001) (Figure 9). *TIMP2* showed positive correlations with *MMP1*, *MMP3*, *MMP8*, *MMP9*, and *MMP16* (*p* < 0.001), and notably, strong positive correlations with *MMP2*, *MMP14*, and MMP13 (*p* < 0.001). *TIMP3* exhibited strong positive correlations with *MMP2*, *MMP13*, *MMP14*, and *MMP16* (*p* < 0.001) (Figure 9). Conversely, *TIMP4* displayed negative correlations with *MMP1*, *MMP9*, and *MMP13* (*p* < 0.001) and positive correlations with *MMP2*, *MMP3*, and *MMP16* (*p* < 0.001) (Figure 9). Most correlations between TIMPs and MMPs were positive, which is particularly interesting given that TIMPs are known inhibitors of MMP activity. These results suggest the existence of transcriptional regulation between TIMPs and MMPs.

### 3.5. Correlation Analysis of TIMPs with ADAMs

**Figure 10:**
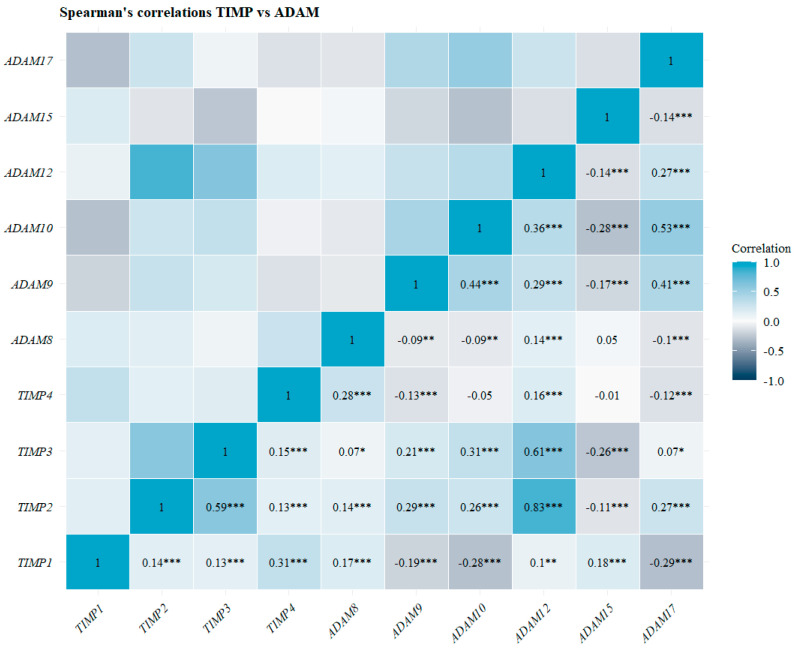
Spearman correlation matrix between TIMPs and ADAMs. Gene expression levels in invasive breast cancer. Results are presented as mRNA expression Z-scores relative to normal samples. Correlations were calculated using the Spearman coefficient with the “Hmisc” package in R (version 4.3.2). Statistical significance levels are reported as *p* < 0.05 (*), *p* < 0.01 (**), and *p* < 0.001 (***). Colors range from blue (positive correlation) to gray (negative correlation).

ADAMs constitute families of proteases that are associated with the development and progression of breast cancer by promoting the shedding of growth factors and receptors from their ectodomains, thereby activating signaling pathways such as EGFR, PI3K/AKT/mTOR, TNF-α, Notch, and JAK-STAT [16]. We performed a Spearman correlation matrix between TIMP and ADAM gene expression levels. Our analysis revealed both positive and negative correlations between TIMPs and ADAMs. *TIMP1* exhibited a positive and statistically significant correlation with *ADAM8*, *ADAM12*, and *ADAM15* and a negative correlation with *ADAM9*, *ADAM10*, and *ADAM17* (*p* < 0.001) (Figure 10). *TIMP2* and *TIMP3* showed statistically significant positive correlations with *ADAM8*, *ADAM9*, *ADAM10*, and *ADAM17*, as well as a strong correlation with *ADAM12* (*p* < 0.05, *p* < 0.01, and *p* < 0.001). Additionally, *TIMP2* and *TIMP3* displayed negative correlations with *ADAM15* (*p* < 0.001) (Figure 10). *TIMP4* showed positive correlations with *ADAM8* and *ADAM12*. Interestingly, *TIMP4* exhibited the strongest negative correlations with ADAMs, including *ADAM9*, *ADAM10*, *ADAM15*, and *ADAM17* (Figure 10).

### 3.6. Association Between TIMP Expression and Immune Cell Infiltration in Breast Cancer

**Figure 11:**
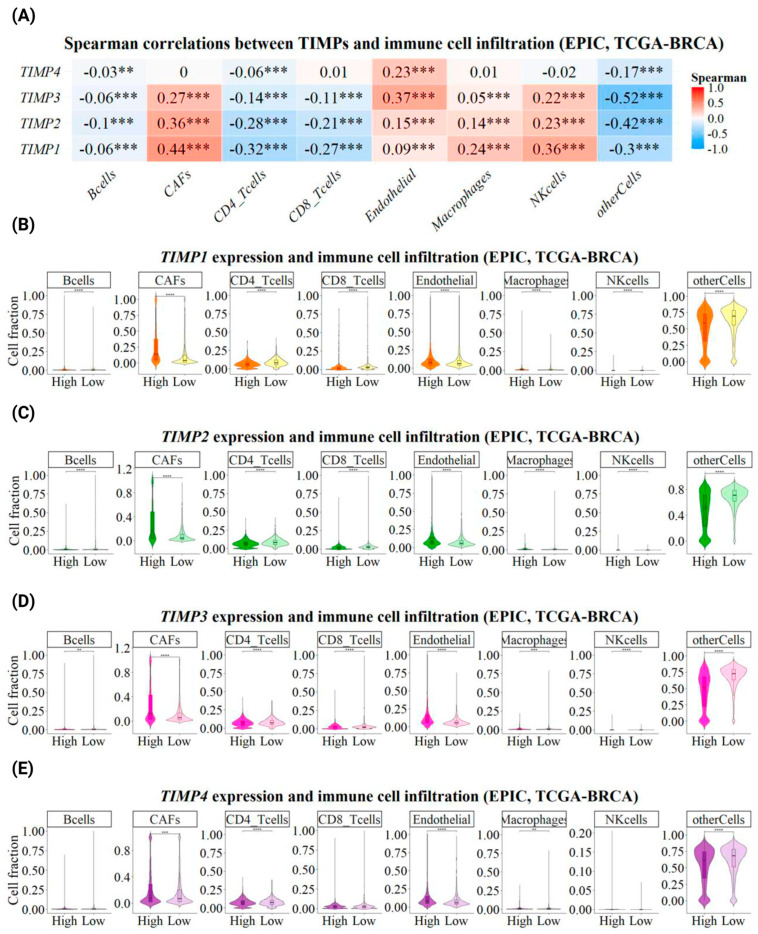
Association between TIMP expression and immune cell infiltration in breast cancer. (**A**) Heatmap showing Spearman correlation coefficients between the expression levels of *TIMP1*, *TIMP2*, *TIMP3*, and *TIMP4* and the estimated infiltration fractions of major immune and stromal cell populations, as inferred by the EPIC algorithm using the TCGA PanCancer Breast Invasive Carcinoma dataset via cBioPortal for Cancer Genomics RNA-seq data. Color intensity represents the direction and magnitude of the correlation (red, positive; blue, negative). Statistical significance is indicated as *p* < 0.01 (**) and *p* < 0.001 (***). (**B**–**E**) Violin plots comparing immune and stromal cell infiltration levels between high- and low-expression groups for *TIMP1* (**B**), *TIMP2* (**C**), *TIMP3* (**D**), |and *TIMP4* (**E**). Patients were stratified based on the median expression value of each TIMP. Estimated cell fractions are shown for B cells, CAFs, CD4^+^ T cells, CD8^+^ T cells, endothelial cells, macrophages, NK cells, and other cells. Statistical differences between groups were assessed using the Wilcoxon rank-sum test with Benjamini–Hochberg false discovery rate correction. Significance levels are indicated as *p* < 0.01 (**), *p* < 0.001 (***), and *p* < 0.0001 (****).

The tumor microenvironment in breast cancer encompasses a wide range of cell populations from both the innate and adaptive immune systems, which have been reported as biologically/clinically relevant to varying degrees [36]. In this context, immune infiltrates have emerged as clinically relevant and highly reproducible biomarkers capable of influencing breast cancer prognosis [37]. In addition to immune cell infiltration, cancer-associated fibroblasts (CAFs) are key players in stromal-dependent multicellular alterations that contribute to cancer initiation and progression [38]. While endothelial cells are involved not only in T-cell trafficking but also in remodeling T-cell function and differentiation [39], the enormous heterogeneity of immune infiltrates makes it inappropriate to group them as a single population. We observed that TIMP expression is related to immune cell infiltration (Figure 11). *TIMP1*, *TIMP2*, and *TIMP3* expressions showed a positive correlation with CAFs, endothelial cells, macrophages, and NK cells (Figure 11A). In contrast, *TIMP4* only showed a positive correlation with endothelial cells (Figure 11A). In contrast, it was observed that the expressions of *TIMP1*, *TIMP2*, *TIMP3*, and *TIMP4* showed a positive correlation with immune infiltrates corresponding to B cells, CD4^+^ T cells, CD8^+^ T cells, and other cells representing the remaining immune cells (Figure 11A). Furthermore, it was found that high and low TIMP expression differentially favored the profile of immune infiltrates (Figure 11B–E). High levels of *TIMP1*, *TIMP2*, *TIMP3*, and *TIMP4* expression were found to favor a greater number of immune infiltrates corresponding to CAFs, endothelial cells, and NK cells. In contrast, high *TIMP1* expression increased B cell and macrophage infiltration. High *TIMP3* expression increased CD4^+^ T cell infiltration. Furthermore, it was observed that low levels of *TIMP1*, *TIMP2*, *TIMP3*, and *TIMP4* expression favored the infiltration of CD8^+^ T cells and other cells. Low expressions of *TIMP2*, *TIMP3*, and *TIMP4* favored the infiltration of B cells and macrophages. Meanwhile, low *TIMP1* expression was observed to increase the infiltration of CD4^+^ T cells.

## 4. Discussion

Worldwide, breast cancer is the most common malignancy and the leading cause of cancer-related deaths in women [1]. Despite advances in breast cancer therapy, it is crucial to identify new biomarkers related to the subtype, grade, and size of breast tumors. TIMPs could be considered therapeutic targets which are involved in the progression and development of breast cancer. In particular, an imbalance between the expression of TIMPs and MMPs has been described as an important factor in cancer development by affecting the integrity of the extracellular matrix [12], inducing apoptosis, and promoting breast cancer migration, invasion, and angiogenesis [9].

TIMP-1 is a multifunctional protein that regulates the activation of different metalloproteinases, including MMPs and ADAMs. It also plays a central role in carcinogenesis, controlling the development and progression of breast cancer [19,40]. Our results show that *TIMP1* mRNA levels are overexpressed in breast cancer patients across different molecular subtypes, specifically in Luminal A and normal-like subtypes. We found that *TIMP1* overexpression tends to be associated with improved overall survival in patients. However, *TIMP1* exhibited positive correlations with enrichment in NF-kappa B signaling, focal adhesions, and PI3K-Akt pathways, which are signaling pathways associated with breast cancer progression. Additionally, *TIMP1* was positively correlated with genes involved in biological processes regulating extracellular matrix organization, inflammatory response, and the positive regulation of cell migration and proliferation, as well as ERK1 and ERK2 signaling, and with components of the actin cytoskeleton and cell surface, which may promote cellular migration and invasion. We also found that *TIMP1* is positively correlated with *MMP2*, *MMP3*, *MMP8*, *MMP9*, and *MMP14*. In this context, previous studies have reported that TIMP-1 regulates EMT [41] in MCF10A mammary epithelial cells through Twist regulation, which suggests that TIMP-1 is upregulated in breast cancer, increasing survival in breast cancer patients [42]. However, it has been reported that high serum and tissue TIMP-1 levels in breast cancer patients are positively associated with lymph node metastasis, lower overall, and relapse-free survival [43]. Another study showed that reduced *TIMP-1* expression in TNBC cell lines inhibited chemoresistance to cisplatin and doxorubicin, thereby inducing cell death [44]. A study that performed univariate and multivariate analyses reported that colorectal cancer patients with high or elevated serum TIMP-1 levels (HR = 1.4; *p* = 0.017) had poorer survival outcomes compared to patients with low or normal serum TIMP-1 levels (HR = 2.2 and 2.1; *p* < 0.001) [45]. In addition, high levels of TIMP-1 are associated with an increased risk of relapse in patients with primary breast carcinoma [46]. After a 3-year follow-up of breast cancer patients, high levels of TIMP-1 and MMP-9 were significantly associated with bone metastasis [47]. High levels of TIMP-1 in breast cancer have been reported to promote growth and inhibit apoptosis through activation of FAK, PI3K, Akt, and Bcl-2 signaling [40,48]. In addition, high levels of TIMP-1 in tumor tissue and serum of breast cancer patients were associated with a low response to chemotherapy and endocrine therapy [49,50]. TIMP-1 also induced the expression of transcription factors that promote EMT, such as TWIST, ZEB1, and ZEB2, thereby inhibiting the expression of epithelial markers and promoting the expression of mesenchymal markers [41]. A previous report showed no association between TIMP-1 polymorphism and breast cancer; however, patients with C/C genotypes reported elevated TIMP-1 levels, suggesting that the C allele may influence the level of TIMP-1 expression in serum [51]. A study demonstrated that TIMP-1 can inhibit MMP-9 and reported that engineered variants of TIMP-1 enhance both affinity and specificity for MMP-9 inhibition through interactions with its catalytic domain and fibronectin domain [52].

Our findings demonstrate that *TIMP1* is significantly overexpressed in Luminal A and normal-like tumors, which is associated with improved overall patient survival. However, TIMP1 may exert a dual, context-dependent role in breast cancer—thus dis-playing either antitumor or protumor effects—as supported by previously reported evidence. Its antitumor activity is related to its ability to inhibit MMPs and ADAMs, whereas its protumor activity is associated with positive correlations with NF-κB, PI3K–Akt, and ERK signaling, as well as focal adhesion signaling, and with biological processes related to extracellular matrix remodeling, inflammation, cell migration, and proliferation.

TIMP-2 participates in the remodeling of the extracellular matrix and can regulate the proliferation, invasion, and chemoresistance of tumor cells [53,54]. Our study found that the mRNA expression level of *TIMP2* was downregulated across the different molecular subtypes of breast cancer. Specifically, *TIMP2* showed lower expression in the Luminal B and basal (TN) molecular subtypes compared to the Luminal subtype. Interestingly, we observed a significant decrease in *TIMP2* expression in stage III compared to stage I. However, *TIMP2* expression was not associated with overall survival in breast cancer patients. In addition, we found that *TIMP2* exhibited a positive correlation with signaling pathways and genes involved in breast cancer progression, migration, and invasion. These included the Hedgehog signaling pathway, adherens junctions, focal adhesions, actin cytoskeleton regulation, and the MAPK signaling pathway, as well as focal adhesion disassembly, actin filament depolarization, stress fiber components, lamellipodia, extracellular matrix components, and actin cytoskeleton elements. Furthermore, we found that *TIMP2* showed positive correlations with *MMP1*, *MMP3*, *MMP8*, *MMP9*, and *MMP16*. Previously, Peney et al. reported that TIMP-2 inhibits TNBC growth and metastasis in a murine model through modulation of epithelial–mesenchymal transition (EMT) via decreased expression of SNAIL, SLUG, ZEB1, and vimentin, as well as inhibition of PI3K and p27 [55]. However, Ree et al. reported that TIMP-2 overexpression correlates with breast cancer progression; notably, they found that high TIMP-2 mRNA levels promote lymph node metastasis in breast cancer patients [56].

Furthermore, high protein levels TIMP-2 in breast cancer tissues correlate with lower overall survival and recurrence-free survival in patients [57]. Interestingly, this dual effect of TIMP-2 is regulated at different stages of carcinogenesis, and the effect varies according to the tumor microenvironment; therefore, it has been suggested that TIMP-2, TIMP-4, MMP-14 and MMP-2 levels should be determined together for prognostic evaluation [58]. In addition, TIMP-2 is a determinant in regulating MMP-2 activation, where TIMP-2 binds to proMMP-2 and forms a complex with MMP-14, which cleaves MMP-2, thus initiating its activation process [59]. These findings suggest that TIMP-2 may have a dual role in mammary carcinogenesis, promoting or inhibiting tumor progression in breast cancer.

TIMP-3 has been described to have anticancer effects, induce apoptosis, and inhibit angiogenesis and metastasis [60,61]. Interestingly, it has been described as a possible biomarker and is the only TIMP that has been proposed as a therapeutic target in cancer [60,61,62]. In contrast, plasma from patients with oral head and neck squamous cell carcinoma has been found to have decreased *TIMP3* mRNA levels compared to healthy patients [61]. It has also been reported that overexpression of TIMP-3 inhibits in vitro invasion and promotes apoptosis of HeLa cervical cancer cells and HT1080 fibrosarcoma [63]. We found that *TIMP3* is downregulated across the molecular subtypes of breast cancer, with a statistically significant decrease observed in the HER2 and basal (TN) subtypes. We found that *TIMP3* expression decreased in stage II and III tumors compared to stage I in breast cancer patients. However, *TIMP3* expression was not significantly associated with overall survival. Positive correlation analysis of *TIMP3* revealed enrichment in Hedgehog signaling, breast cancer-related signaling pathways, cell adhesion molecules, focal adhesions, and PI3K-Akt signaling. Additionally, *TIMP3* was positively correlated with genes encoding extracellular matrix components, including collagen trimers, membrane coverings, and adherens junctions, as well as genes with molecular functions related to extracellular matrix constituents that confer elasticity and microtubule-binding proteins. Furthermore, *TIMP3* displayed strong positive correlations with *MMP2*, *MMP13*, *MMP14*, and *MMP16.* Previous reports indicate that decreases in TIMP-3 levels are due to hypermethylation of the TIMP3 gene promoter and transcriptional repression. This reduces TIMP-3’s ability to inhibit the activity of MMPs, which consequently allows cancer cells to adopt a malignant and invasive phenotype [64,65]. In 1996, Bian, J. et al. reported that overexpression of *TIMP-3* in colon carcinoma DLD-1 cells inhibited cell growth and inhibited tumor-forming capacity in nude mice [66]. In another study, they reported that decreased TIMP-3 expression increased interleukin-6 (IL-6) production, which promoted cell growth and invasion and decreased overall survival and relapse-free survival periods in HPV-infected non-small-cell lung cancer patients [67]. In contrast, in an in vivo study using mice, they reported that silencing of TIMP-3 resulted in the suppression of mammary tumors and suggested that it was under the regulation of the *Tnf* or *Tnfr1* genes [68]. It has also been reported that breast cancer patients with high TIMP-3 mRNA levels are associated with a better response to endocrine therapy with tamoxifen [69]. Another in vitro study using the breast cancer cell lines HEK293T, MCF-7, T47D, and MDA-MB-231 demonstrated that HBP1 enhanced the activity of the TIMP3 promoter, and TIMP3 inhibited the ubiquitination of the PTEN protein, thereby preventing its degradation and suppressing the p-AKT signaling pathway. Furthermore, TIMP3 was found to inhibit cell proliferation and invasion by downregulating the expression of N-cadherin, MMP2, and MMP9. In vivo, the HBP1/TIMP3 axis was reported to suppress breast cancer metastasis [70]. There is evidence suggesting that TIMP-3 exerts antimetastatic effects by inhibiting MMPs and ADAMs, reducing angiogenesis, suppressing tumor growth, and inducing apoptosis through the stabilization of death receptors [71]. Interestingly, in biopsies of patients with head and neck carcinoma, overexpression of TIMP-3 mRNA was associated with poorer survival compared to those with low mRNA levels [72]. Interestingly, results in other types of cancer indicated that TIMP3 overexpression was associated with shorter survival. In contrast, in our study, we report that lower TIMP3 expression was associated with decreased survival in breast cancer patients. Our results suggest that TIMP3 exhibits antitumor activity and may be involved in the inhibition of breast cancer metastasis by suppressing MMPs and ADAMs, inhibiting PI3K-Akt signaling, reducing angiogenesis, and inducing apoptosis. However, this effect is likely to depend on the molecular subtype of breast cancer and on epigenetic silencing mechanisms.

TIMP-4 is the least studied member of the TIMPs; however, elevated levels of TIMP-4 have been found in breast, ovarian, cervical, prostate, brain, colon, endometrial, and renal papillary tumors. In contrast, downregulation was observed in pancreatic and renal clear cell tumors [73]. In tumor tissues from patients with oral squamous cell carcinoma and head and neck squamous cell carcinoma, a significant decrease in *TIMP4* expression was observed compared to normal tissue. Furthermore, Kaplan–Meier analysis showed that patients with high *TIMP4* expression had longer survival times and higher survival rates [74]. However, our results show that *TIMP4* is downregulated across the molecular subtypes of breast cancer, with a statistically significant decrease observed in the Luminal B, HER2, and basal (TN) subtypes. Furthermore, *TIMP4* expression was significantly reduced in stage II tumors compared to stage I in breast cancer patients. Interestingly, there was a trend toward higher overall survival in patients with low *TIMP4* expression.

Functional enrichment analysis using KEGG revealed that significantly enriched signaling pathways included VEGF signaling, resistance to EGFR tyrosine kinase inhibitors, breast cancer-related signaling pathways, MAPK signaling, and PI3K-Akt signaling. Additionally, positive correlations were observed between *TIMP4* and genes involved in biological processes, cellular components, and extracellular matrix constituents that confer elasticity, kinase activity, and DNA-binding transcription factor activity. Background reports indicate that in nude mice with cervical cancer cell xenografts, overexpression of TIMP-4 induces tumor formation. In vitro, assays promote the enrichment of the cancer stem cell population through NFκB signaling [75]. In contrast, they report that in patients with estrogen receptor-negative infiltrating ductal breast cancer, overexpression of TIMP-4 is associated with malignant progression and correlates with a low probability of long-term disease-free survival (more than 3 years) [76]. TIMP-4 also inhibited apoptosis of MDA-MB-435 breast cancer cells in vitro and in vivo employing a nude mouse model by regulating the expression of TIMP-4, which upregulates the expression of Bcl-2 and Bcl-xL [77].

The antitumor effect of *TIMP4* may be related to its ability to inhibit MMPs. Our analysis showed that *TIMP4* was positively correlated with *MMP2*, *MMP3*, *MMP8*, *MMP14*, and *MMP16*; interestingly, it was the only TIMP that exhibited a negative correlation with *MMP1*, *MMP9*, and *MMP13*. Our results are consistent with a study in which breast cancer patients treated with radiotherapy exhibited positive correlations between *TIMP4* and *MMP2*, *MMP3*, *MMP7*, *MMP8*, and *MMP9*, and the authors suggested that *TIMP4* could be used as a prognostic and predictive biomarker for breast cancer patients undergoing radiotherapy [33].

ADAMs have been reported to induce proliferation, tumor growth, migration, invasion, and metastasis in breast cancer [78]. TIMPs can stabilize and modulate ADAM-mediated signaling [8]. Our analysis showed that *TIMP1* was positively correlated with *ADAM8*, *ADAM12*, and *ADAM15*, and negatively correlated with *ADAM9*, *ADAM10*, and *ADAM17*. Interestingly, we found a strong positive correlation between *TIMP2* and *TIMP3* with *ADAM12*, as well as between *TIMP2* and *TIMP3* and *ADAM10*, and negative correlations with *ADAM15*. In contrast, *TIMP4* was positively correlated with *ADAM8* and *ADAM12* and negatively correlated with *ADAM9*, *ADAM10*, *ADAM15*, and *ADAM17*. These findings are noteworthy because these proteins have been associated with invasive phenotypes, epithelial–mesenchymal transition (EMT), and breast cancer metastasis. Together, our results and previous experimental evidence support the notion that TIMP4 may exert a pro-tumorigenic role depending on the breast cancer context and other cancer types, through its positive association with oncogenic signaling pathways such as VEGF, PI3K–Akt, MAPK, and EGFR.

Data indicate that immune infiltrates are considered clinically relevant biological actors and biomarkers capable of affecting the prognosis of breast cancer [36]. It has been reported that TN breast cancer subtypes are more likely to present with tumors exhibiting >50% lymphocytic infiltration and have the greatest survival benefit for each 10% increase in lymphocytes [79]. Most HER2^+^ breast cancers have a similar level of immune infiltration to TN breast cancer; however, the presence of CD4^+^ T cell infiltrates has not demonstrated the same survival benefit. In contrast, HER2-negative, hormone receptor-positive subtypes tend to exhibit the least immune infiltration but are the only breast cancer subtype that shows a worse prognosis with greater T-cell infiltration [79]. NK cells play a crucial role in regulating anticancer activity and are a favorable prognostic factor in breast cancer [80]. Macrophages are highly heterogeneous and the most abundant immune cells in the tumor microenvironment, specifically in the tumor-adipose microenvironment (TAME) [81]. Increased macrophages in breast tissue associated with TAME have been reported to predict a poor prognosis for breast cancer [81]. Similarly, B lymphocytes have been observed to promote tumor progression by affecting various cell types, such as T lymphocytes and macrophages in cancer [82]. In addition to immune cell infiltration, CAF cells are key players in stromal-dependent multicellular alterations that contribute to cancer initiation and progression [38]. Tumor-associated endothelial cell infiltrates also play a significant role, as they are known to be related to tumor growth and metastasis [83]. In our study, we found that the expression levels of *TIMP1*, *TIMP2*, and *TIMP3* showed a positive correlation with CAFs, endothelial cells, macrophages, and NK cells. *TIMP4* showed a positive correlation exclusively with endothelial cells. Meanwhile, the expression levels of *TIMP1*, *TIMP2*, *TIMP3*, and *TIMP4* showed a positive correlation with immune infiltrates corresponding to B cells, CD4^+^ T cells, CD8^+^ T cells, and other cells representing the remaining immune cells. Furthermore, high and low *TIMP* expression differentially favored the profile of immune infiltrates. The study found that high levels of *TIMP1*, *TIMP2*, *TIMP3*, and *TIMP4* expression favored a greater number of immune infiltrates, including CAFs, endothelial cells, and NK cells. High *TIMP1* expression increased B cell and macrophage infiltration. High *TIMP3* expression increased CD4^+^ T cell infiltration. Conversely, low levels of *TIMP1*, *TIMP2*, *TIMP3*, and *TIMP4* expression favored CD8^+^ T cell and other cell types. Low *TIMP2*, *TIMP3*, and *TIMP4* expression favored B cell and macrophage infiltration; however, low *TIMP1* expression was associated with increased CD4^+^ T cell infiltration. These results suggest that high expression levels of *TIMP1*, *TIMP2*, *TIMP3*, and *TIMP4* promote greater infiltration of CAFs, macrophages, endothelial cells, and B cells, which may regulate tumor progression and contribute to poor survival in breast cancer patients. In contrast, low TIMP expression—specifically, of *TIMP1*, *TIMP2*, *TIMP3*, and *TIMP4*—promotes greater infiltration of CD4^+^ T lymphocytes, CD8^+^ T lymphocytes, and other cellular infiltrates, which may be associated with better treatment response, improved prognosis, and more favorable survival in breast cancer patients. However, to validate the findings of our analysis, further experimental studies are required to more robustly support these observations and to elucidate the molecular effects of TIMPs in the context of breast cancer, as well as their potential utility as prognostic biomarkers and/or therapeutic targets.

Despite the robustness of the datasets analyzed and the consistency of the associations observed across multiple independent cohorts, this study is inherently exploratory and presents limitations that should be acknowledged. First, the present work is predominantly bioinformatic in nature and is based on retrospective analyses of publicly available transcriptomic datasets, which precludes direct causal inference or mechanistic validation. The correlations identified among TIMPs, MMPs, ADAMs, signaling pathways, and immune or stromal cell infiltration reflect associative relationships rather than direct regulatory effects. Second, although external validation was performed using GEO datasets and protein-level information from the Human Protein Atlas, no functional experimental validation was conducted. Consequently, the proposed biological roles of TIMPs in breast cancer progression, tumor microenvironment modulation, and immune infiltration should be interpreted as hypothesis-generating rather than definitive. In addition, immune and stromal cell infiltration was inferred using computational deconvolution (EPIC), which estimates relative cellular proportions from bulk RNA sequencing data and does not fully capture intratumoral heterogeneity or the spatial organization within the tumor microenvironment. Finally, although the survival analyses detected robust population-level trends and identified potential prognostic signals for TIMPs in breast cancer, they were performed using unadjusted models and may be influenced by clinical and treatment-related confounding factors that are not uniformly available across the different datasets.

Taken together, these limitations highlight the need for future experimental and prospective studies to validate the molecular interactions and biological effects suggested by our analyses. Nevertheless, the integrative bioinformatic approach employed here provides a comprehensive and systematic framework that identifies clinically relevant TIMP-associated signatures and generates biologically meaningful hypotheses for further mechanistic investigation in breast cancer.

## 5. Conclusions

Our results revealed that TIMP1 was the only TIMP overexpressed in tumor samples from breast cancer patients, with higher expression observed in the more aggressive molecular subtypes. In contrast, TIMP2, TIMP3, and TIMP4 were downregulated in tumor samples and across the molecular subtypes of breast cancer. Interestingly, high expression of TIMP1 and TIMP4 showed a trend toward improved overall survival. Functional enrichment analyses (KEGG and GO) of genes positively correlated with TIMP1 and TIMP2 revealed associations with processes involved in the regulation of the tumor microenvironment, potentially contributing to breast cancer cell migration and invasion. TIMP3 and TIMP4 were associated with genes involved in cytoskeletal regulation and DNA damage response signaling. Furthermore, TIMPs exhibited both positive and negative correlations with MMPs and ADAMs, suggesting the existence of autoregulatory mechanisms between these proteins. High expression of TIMP1, TIMP2, TIMP3, and TIMP4 favors greater infiltration of CAF cells, macrophages, endothelial cells, and B cells. Conversely, low expression of TIMP1, TIMP2, TIMP3, and TIMP4 favors greater infiltration of CD4^+^ and CD8^+^ T lymphocytes, demonstrating that differential TIMP expression is related to progression and a poorer prognosis in breast cancer patients.

## Data Availability

The datasets used in this research were obtained from the cBioPortal for Cancer Genomics database “http://cbioportal.org (10 November 2025)” the Breast Invasive Carcinoma TCGA PanCancer dataset, Gene Expression Omnibus GEO “https://www.ncbi.nlm.nih.gov/geo/ (4 January 2026)” and The Human Protein Atlas “https://www.proteinatlas.org/ (6 January 2026)”. Additional data generated in this study are available from the corresponding author upon request.

## Abbreviations

The following abbreviations are used in this manuscript:

TIMPs	Tissue Inhibitor of Metalloproteinase
TIMP1, TIMP2, TIMP3, TIMP4	Tissue Inhibitors of Metalloproteinases1, 2, 3, and 4
HER2	Human Epidermal Growth Factor Receptor 2
TNBC	Triple-Negative Breast Cancer
MMPs	Matrix Metalloproteinases
MMP1, MMP2, MMP3, MMP7, MMP8, MMP9, MMP13, MMP14, MMP16	Matrix Metalloproteinases 1, 2,3, 7, 8, 9, 13, 14 and 16
ER	Estrogen Receptors
PR	Progesterone Receptors
TNM	Tumor/Node/Metastasis
ECM	Extracellular Matrix
ADAM	A Disintegrin and Metalloprotease
AJCC	American Joint Committee on Cancer
KEGG	Kyoto Encyclopedia of Genes and Genomes
GO	Gene Ontology
OS	Overall Survival
BP	Biological Processes
CC	Cellular Components
MF	Molecular Function
